# The Nematicidal Effect of *Camellia* Seed Cake on Root-Knot Nematode *Meloidogyne javanica* of Banana

**DOI:** 10.1371/journal.pone.0119700

**Published:** 2015-04-07

**Authors:** Xiujuan Yang, Xuan Wang, Kang Wang, Lanxi Su, Hongmei Li, Rong Li, Qirong Shen

**Affiliations:** 1 Jiangsu Key Lab and Engineering Center for Solid Organic Waste Utilization, National Engineering Research Center for Organic-based Fertilizers, Jiangsu Collaborative Innovation Center for Solid Organic Waste Resource Utilization, Nanjing Agricultural University, Nanjing, China; 2 Key Laboratory of Integrated Management of Crop Diseases and Pests, Ministry of Education, Department of Plant Pathology, Nanjing Agricultural University, Nanjing, China; CEA (Atomic and Alternative Energies Commission), FRANCE

## Abstract

Suppression of root-knot nematodes is crucially important for maintaining the worldwide development of the banana industry. Growing concerns about human and environmental safety have led to the withdrawal of commonly used nematicides and soil fumigants, thus motivating the development of alternative nematode management strategies. In this study, *Meloidogyne javanica* was isolated, and the nematicidal effect of *Camellia* seed cake on this pest was investigated. The results showed that in dish experiments, *Camellia* seed cake extracts under low concentration (2 g/L) showed a strong nematicidal effect. After treatment for 72 h, the eggs of *M*. *javanica* were gradually dissolved, and the intestine of the juveniles gradually became indistinct. Nematicidal compounds, including saponins identified by HPLC-ESI-MS and 8 types of volatile compounds identified by GC-MS, exhibited effective nematicidal activities, especially 4-methylphenol. The pot experiments demonstrated that the application of *Camellia* seed cake suppressed *M*. *javanica*, and promoted the banana plant growth. This study explored an effective nematicidal agent for application in soil and revealed its potential mechanism of nematode suppression.

## Introduction

Bananas (*Musa* spp.) are among the most important crops in the world as a staple food, and they are also the main source of income for local farmers in many developing countries [1]. However, the production of bananas is hampered by many diseases and pests [2]. Among the most damaging banana pests are the widespread plant-parasitic nematodes. In particular, the root-knot nematodes, *Meloidogyne* spp., are economically important soil-borne pathogens that are reported to infect almost all of the world’s major crop plants [3]. Infection with root-knot nematodes causes root damage that not only leads to severe crop losses in commercial banana plantations for export but also seriously limits the production and viability of other banana types [4]. *M*. *javanica*, one species of the root-knot nematodes, attacks banana plants at an early stage in the field [5] and induces the root cells into giant cells. Through these giant cells, the root-knot nematode sucks up nutrients and prevents the plant growth, thereby results the symptom of stunting and yellowing. Thus, it is both necessary and urgent to find a better way to suppress this harmful nematode and maintain worldwide development of the banana industry.

Nematode management is traditionally achieved by application of nematicides. However, growing concerns about human and environmental safety have led to the withdrawal of several commonly used nematicides and soil fumigants [6]. In a previous study, 55 banana accessions were evaluated for resistance to nematode species [7], but no source of resistance was found. Soil fumigation as a pre-plant treatment is reported to be effective in suppressing the nematode population in some plants [8]; however, the effect is short-lived compared to the life of a banana crop. Moreover, plant-parasitic nematodes tend to repopulate an area fairly quickly after fumigation, and some fumigants are potential environmental contaminants [9]. These findings motivate the development of alternative nematode management strategies.

Effective control strategies for *M*. *javanica* may include the use of plant-origin nematicidal agents [10]. *Camellia* seed cake is an organic substance made of the residual from *Camellia oleifera* Abel seeds after oil extraction. The saponin released from this by-product can kill muciferous mollusks [11] and could therefore be used to prevent the damage caused by root-knot nematodes. Oil cakes in combination with *Bradyrhizobium* sp. and *Paecilomyces lilacinus* have already been studied for the control of mungbean root knot nematode [12]. Tea-oil *Camellia* seed cake extracted with methanol or water was tested for its effects on controlling *Bursaphelenchus xylophilus* [13]. Because of the high nutritional value and essential elements together with mammalians safety, the tea-oil *Camellia* seed cake using as a non-conventional fertilizer is increasingly gaining priority [14–15]. However, to our knowledge, the nematicidal effects of *Camellia* seed cake and its potential mechanisms have not yet been studied. In particular, no reports have described the effects of using *Camellia* seed cake to amend banana-planting soil for the suppression of *M*. *javanica*.

In this study, the nematicidal ability of *Camellia* seed cake was investigated in detail to explore a green strategy for managing *M*. *javanica* in banana plantings. In addition, the nematicidal substance extracted from tea-oil *Camellia* seed cake by ultrapure water was identified using HPLC-ESI-MS, and the nematicidal ability of 8 VOCs (volatile organic compounds) detected using GC-MS were confirmed. This paper highlights how tea-oil *Camellia* seed cake can be used to manage *M*. *javanica* to reduce its harmful effects on banana plants and examines some of the potential mechanisms involved in the control of *M*. *javanica* by tea-oil *Camellia* seed cake.

## Materials and Methods

### Ethics statement

Our study was carried out on the farmers' land (18°23′ N, 108°44′ E) with property rights in China (1996–2035) and farmer Yusheng Li should be contacted for future permissions. No specific permits were required for the described field studies and the locations are not protected. The field studied did not involve endangered or protected species.

### Isolation and identification of *M*. *javanica*

Individual seedlings of *Musa* AAA *Cavendish* cv. Brazil were taken from the field in Ledong, Hainan province, China. Tweezers were used to collect all visible suspect *Meloidogyne* spp. eggmasses after cleaning the roots with deionized water. Then, all the eggmasses (18 in total) were put into plates (one in each plate, numbered C1 to C18) with 5 mL water and hatched at 25°C for 72 h to obtain second-stage juveniles (J2s). Then, the J2s (10 from each plate) were taken for molecular identification *via* species-specific primer pairs [16]. DNA samples of J2s were prepared according to Li et al. 2008 [17]. Primers Far/Rar, Finc/Rinc, JMV1/JMV2 and Fjav/Rjav used for PCR amplification were synthesized by Invitrogen (Shanghai, China).

The J2s of other suspect *Meloidogyne* spp. from each plate were inoculated onto banana roots to produce disease. Banana seedlings were cultured in a seedling matrix that was sterilized at 121°C for 30 min prior to use so that no nematodes were observed in the plant roots. Pot experiments were carried out in a greenhouse located at Hainan Wan Zhong Co., Ltd., Hainan, China. The temperature and relative humidity were in accordance with the environment, and the season was suitable for banana planting. Each pot containing 500 g sterilized soil and banana roots were inoculated with 100 J2s. Banana plants grown in soil with no inoculation were treated as the control (CK).

After 6 months, disease symptoms of plants were observed, the root knots were sampled, and the nematode eggmasses and females were harvested for identification. Nematodes inside banana roots were stained using the sodium hypochlorite acid fuchsin method [18] and observed using an Olympus ZX10 stereo microscope. The selected females of *Meloidogyne* spp. were determined by observation of perineal patterns using an Olympus BX51 microscope (Japan, CCD DP72). Meanwhile, the J2 from one of *Meloidogyne* spp. in plate C11 was selected for further molecular identification. PCR products were cloned into the pMD19-T vector (TaKaRa) and transformed into competent *Escherichia coli* DH5α cells. The 18S rRNA gene sequence was sequenced, and the molecular phylogenetic analysis was performed using MEGA version 4.0 [17].

### Nematicidal effect of Camellia seed cake extracts

*Camellia* seed cake was soaked in sterile ultrapure water in different concentrations (1, 1.5, 2, 2.5, 3, 4, 5, 6, 7, 8, 9, 10, 15, 20, 30, 50, 100 g/L) for 72 h. After filtration, 10 mL of extracts with different concentrations were transferred to 6 cm petri dishes (300 individual freshly hatched J2s of *M*. *javanica* per dish) in order to evaluate their nematicidal effects. An equal volume of deionized water was used as a control. The dishes were allowed to incubate for 36 h, and then the nematodes were observed with an Olympus ZX10 stereo microscope in order to calculate the corrected mortality. Nematodes were considered alive if they moved or appeared as a winding shape and were considered dead if they did not move when probed with a fine needle [19]. Then, the nematodes in each treatment were transferred to distilled water for 48 h to ascertain whether the dead nematodes regained mobility or not. The corrected mortality was calculated according to the following formula: mortality (%) = (mortality of treatment-mortality of CK) / (1-mortality of CK) × 100. Morphological variations of *M*. *javanica* juveniles during 72 h treatment with 5 g/L extract were observed using an Olympus BX51 microscope.

To evaluate the effects of *Camellia* seed cake extract on the relative juveniles hatching ratio, eggmasses contained total about 500 eggs were inoculated in dishes with 5 g/L, 2 g/L and 1 g/L extract and hatched at 25°C for 120 h. Each extract has 5 replicates. Hatching J2s were observed with an Olympus ZX10 stereo microscope. The hatching ratio was calculated at 24 h intervals as (%) = (incubation of treatment-incubation of CK) / (1-incubation of CK) × 100. Morphological variations of eggs treated with 5 g/L extract for 72 h were observed using an Olympus BX51 microscope.

### Nematicidal effect of the extracted camellia saponin

The camellia saponin was extracted according to the method of Zhong et al. [20]. Briefly, 13.25 g of *Camellia* seed cake was ground, wrapped in a filter paper package and placed in a drying oven maintained at 70°C for 2 h to remove water. Then, extraction proceeded using 200 mL methanol (HPLC grade) in a soxhlet extractor. Reflux extraction was performed at 5 min per cycle for 4 h. The extract was then evaporated under reduced pressure using a centrifugal evaporator at room temperature. Residual organic material was re-dissolved in a total of 2 mL of methanol. The re-dissolved extract was diluted to different concentrations (6, 7, 8, 9, 10, 13, 15, 20, 30, 50, 70, 100 g/L). Effects of the extracts on the *M*. *javanica* J2s were tested as previously described.

The molecular weight of the saponin was detected by HPLC-ESI-MS (HPLC: 1200 series; ESI-MS: 6410 Triple Quad LC/MS, Agilent, USA) using a C18 column (250 mm × 4.6 mm, 5 μm) as a chromatographic column at a flow-rate of 0.5 mL/min. The column temperature was 20°C, injection volume was 10 μL and wave length for all detections was 280 nm. Mobile phase A was water with 0.1% acetic acid, and mobile phase B was acetonitrile. An elution gradient from 20% to 15% mobile phase A volume fraction, over a gradient time of 0 to 20 min, was used. MS analysis was performed by electrospray ionization in the positive ion mode.

### Nematicidal effect of the volatile compounds of Camellia seed cake

The split plates (Petri dish with vents, Greiner company, Germany) were used to test the nematicidal activity of the volatiles produced by the *Camellia* seed cake. One side of the split plate was tiled with 5 mL sterile ultrapure water with different concentrations of *Camellia* seed cake (10, 12, 15, 20, 30, 50, 100, 150, 200 g/L), and the other side held 5 mL of ultrapure water with 300 individuals of freshly hatched juveniles of *M*. *javanica* (S1 Fig.). An equal volume of ultrapure water was used as a control. Corrected mortality was determined after the plates stood for 72 h.

The volatiles produced by the *Camellia* seed cake extract were collected using the solid-phase micro-extraction (SPME) technique [21], which detects volatiles produced at the headspace of the serum bottle. The SPME syringe, equipped with fiber material (50/30 DVB/Carboxen on PDMS stable flex fiber) [22], was then inserted into the center of the parafilm covering the serum bottle. Fiber material was exposed to the volatiles in the headspace to entrap the volatiles. Three days later, the syringe containing the volatiles was inserted into a Gas Chromatography-Mass Spectrometer (GC-MS). GC-MS analysis was performed in electron ionization (EI) mode (70 eV) using a Finnigan gas chromatograph equipped with an MS detector. A Finnigan capillary column (15 m length × 0.5 mm id × 0.25 mm film thickness) was first used, with the following temperature program: the temperature was held at 35°C for 3 min, then increased to 180°C at 10°C/min; this temperature was held for 1 min, and finally increased to 240°C at 40°C min^-1^ and held at 240°C for 10 min. Helium was used as the carrier gas at a constant flow rate of 1.0 mL/min. The samples were analyzed in split mode (1: 20) with an injection and EI source temperature of 220°C and then scanned in the mass range from 30 m/z to 650 m/z. The compounds produced by *Camellia* seed cake were identified using the NIST (National Institute of Standards and Technology) database on the mass spectrometer.

The nematicidal activity of the various VOCs identified by GC-MS was examined using pure standard substances and split plates. All the selected substances were dissolved with ethanol and later diluted with the sterile ultrapure water. The concentration of diluted solvents was 3000, 300, 30 and 3 mg/L, respectively. Nematicidal activity was tested by placing sterile filter paper discs with 100 μL of diluted solvent in one side of the split plates and 5 mL water with 300 freshly hatched J2s was in another side. The 100 *μ*L of water diluted ethanol was as the control. The corrected mortality of each treatment was determined after standing for 72 h.

### Pot experiments

Pot experiments were performed in a greenhouse located at Hainan Wan Zhong Co., Ltd., Hainan, China, during three banana growing seasons from May to July 2012, September to October 2012 and May to July 2013. Banana seedlings (*Musa* AAA *Cavendish* cv. Brazil) without nematodes in their roots were used in this experiment. The soil for the pot experiments was collected from a field with serious root-knot nematode-wilt disease in Hainan province, China. The soil had a pH value of 6.52, an organic matter content of 10.12 g/kg, and available N, P, K contents of 38.39, 202.19, and 174.22 mg/kg, respectively.

In each season of pot experiments, four treatments were designed as follows. In the control, CK, no *Camellia* seed cake was added to the soil. In treatment A, 5 g/kg of *Camellia* seed cake was added to the soil. In treatment B, 2 g/kg of *Camellia* seed cake was added to the soil. In treatment C, 1 g/kg of *Camellia* seed cake was added to the soil. Each treatment was supplemented with identical nutrient content. Each treatment had three blocks; each block contained nine pots.

In the first-season of pot experiments, the soil for all pots (3 kg soil for each pot) was first sterilized. Then, juveniles of *M*. *javanica* were added to each pot (600 individuals per 1 kg soil). Then, *Camellia* seed cake was added to the pot. Seven days later, one banana seedling free of nematodes was transplanted to the pot. All pots were kept in greenhouse under room temperature for 60 days and the seedlings were removed for the measurement of agronomic characteristics and biomasses. The number of eggmasses on roots was also calculated.

In the second- and third-season of pot experiments, the crude soil with nematodes was put into pot and *Camellia* seed cake was added. Again seven days later, one banana seedling free of nematodes was transplanted to the pot. The conditions for keeping seedlings were the same as described above. Sixty days later, the seedlings were removed from the pots and the agronomic characteristics and biomasses were measured. The roots of each seedling were macerated in a blender and root-knot nematodes were extracted by the Baermann funnel and calculated using an Olympus ZX10 stereo microscope. The total nematodes in soil of each pot were extracted by the Baermann funnel and calculated. The nematodes species were determined using morphological and feeding-habit based classifications. The number of culturable microorganisms including bacteria, fungi and actinomycetes in soil of each pot was determined [23].

### Statistical analysis

Differences among treatments were assessed using a one-way ANOVA analysis. All means were calculated from the values of five replicates and subjected to Duncan’s multiple range tests at *P* = 0.05 and the statistical analysis was carried out using software SPSS version 17.0 (SPSS Inc., Chicago, IL). Different letters indicated in figures means the statistically significant differences at the 0.05 probability level according to the Duncan test.

## Results

### Isolation and identification of *M*. *javanica*

**Fig 1:**
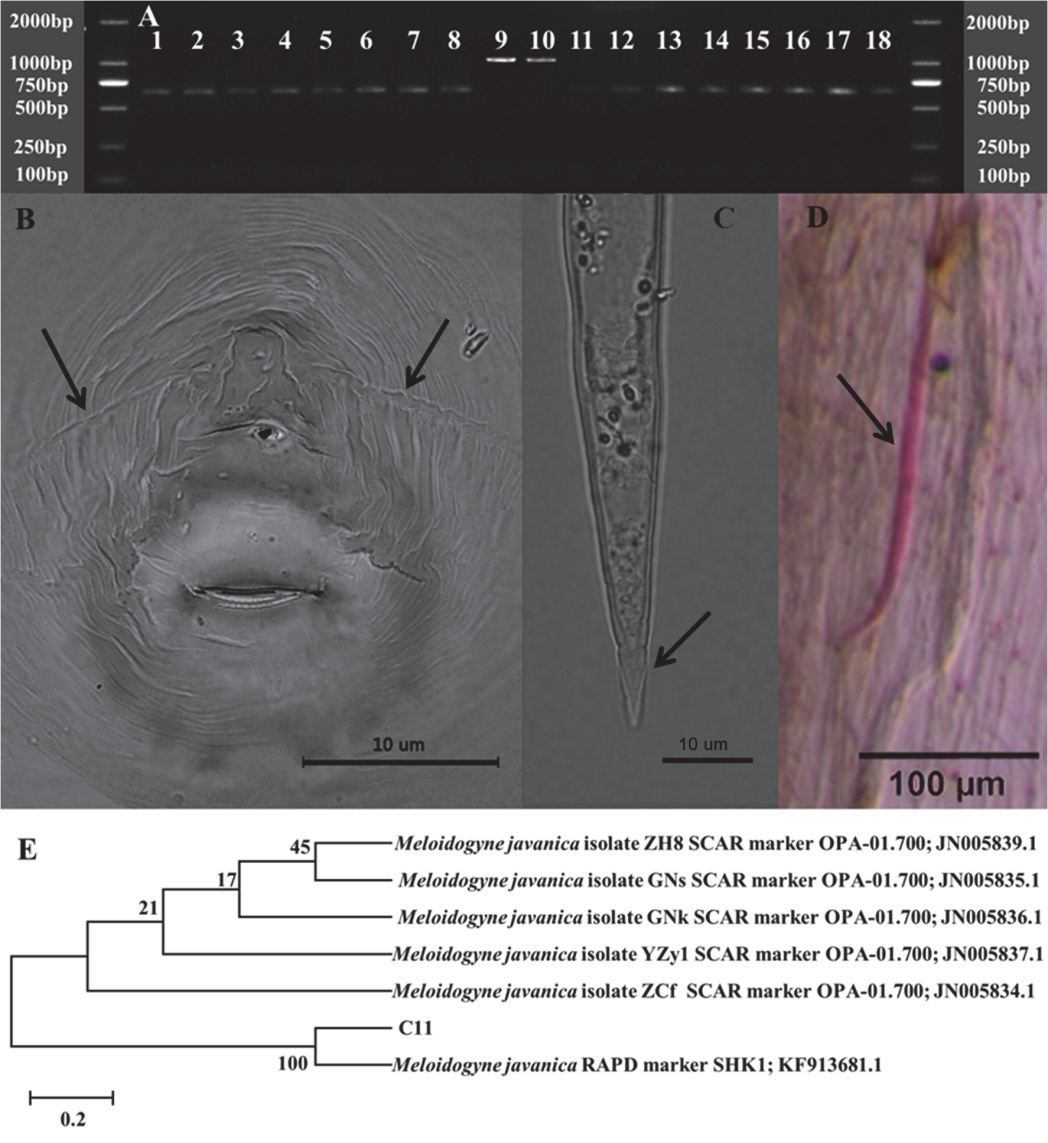
Molecular identification of *M*. *javanica* detected by primers Fjav/Rjav and *M*. *incognita* by Finc/Rinc (A); the female perineal pattern of *M*. *javanica* showing two obvious lateral lines (B); the hyaline tail observed in J2 of *M*. *javanica* (C); the infection of J2s from C11 of *M*. *javanica* in banana roots (D); phylogenetic tree of the sequence of nematode C11 (E). Bars: B, C 10 μm; D 100 μm.

To isolate *M*. *javanica*, 18 individual suspect eggmasses with or without females were first isolated and numbered C1 to C18. All the eggmasses were then hatched into *Meloidogyne* spp. J2s for molecular identification. No PCR products were amplified from all DNA templates using the primers Far/Rar and JMV1/JMV2 (data no shown). Two bands at approximately 1200 bp were observed using the primer Finc/Rinc for C9 and C10; these nematodes were therefore preliminarily identified as *M*. *incognita* [16]. Approximately 670 bp band was detected in the rest of other J2s using the primer Fjav/Rjav (Fig. 1A); these nematodes were therefore preliminarily identified as *M*. *javanica* [16], which confirmed to be the dominant species on banana.

After identification, all the hatched juveniles of *M*. *javanica* were inoculated into the banana plants for disease production. Six months after transplanting, the plants were dwarfed and the leaves were pale, and significant root knots were observed in the treatment plants. The root knots were sampled, and white females were harvested. Two obvious lateral lines were observed in the perineal patterns (Fig. 1B) of the females, and the hyaline tail of juvenile was shown in Fig. 1C. The disease symptoms increased with increasing numbers of inoculated juveniles, and no root knots were observed in the control. The infection of C11 juvenile to banana root is shown in Fig. 1D. Due to C11 has the highest activities for juveniles hatching and root infection than others, therefore, it was further selected for molecular identification. An approximately 670 bp band from primer Fjav/Rjav was amplified and sequenced. The phylogenetic tree of the sequence showed that nematode C11 was related to the *M*. *javanica* lineage and closely clustered with similar species (Fig. 1E). Based on the above phenotypic characteristics and phylogenetic analysis, nematode C11 was confirmed to be *M*. *javanica*. The combination of these results verified that the isolated *M*. *javanica* C11 is from the previous inoculation; and was selected for further study.

### The nematicidal activity of the Camellia seed cake extracts

**Fig 2:**
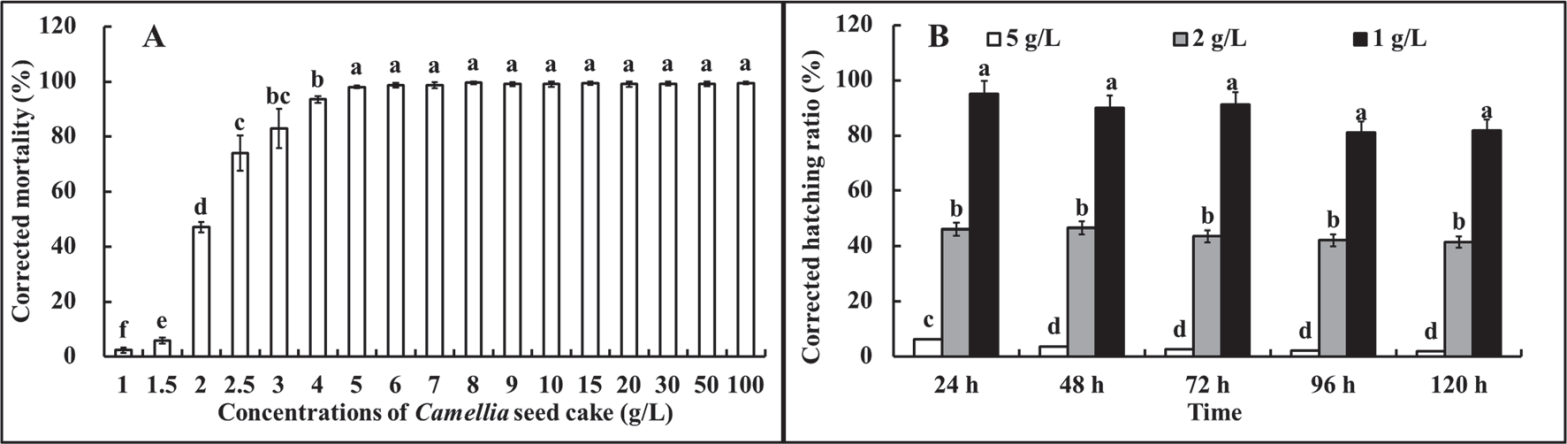
Effects of different concentrations of *Camellia* seed cake extract on the corrected mortality of *M*. *javanica* J2s (A); and the corrected hatching ratio of eggs (B).

A juvenile mortality of approximately 99% was observed for 100 g/L to 5 g/L of *Camellia* seed cake extracts. The rate showed a continual decline from 5 g/L to 1 g/L and tended to be lowest for 1 g/L, at approximately 2.4%. Moreover, the nematicidal rate was 57.11% for 2 g/L of *Camellia* seed cake extract (Fig. 2A). Therefore, concentrations of 5 g/L, 2 g/L and 1 g/L were selected for further study.

Approximately no juveniles were hatched from 0 to 72 h at 5 g/L. At 2 g/L, the hatching ratio was nearly 50% from 24 to 120 h, and at 1 g/L, no inhibition effect was observed compared to the control (Fig. 2B).

**Fig 3:**
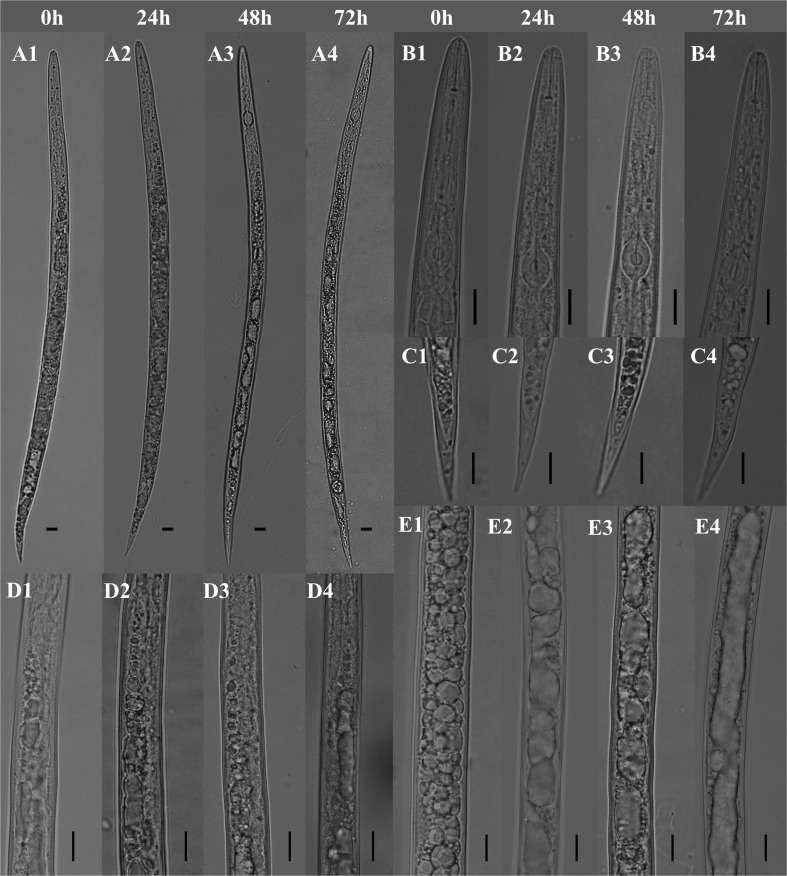
Morphological variations of *M*. *javanica* J2s (the whole body (A), anterior part of the body (B), tail region (C), junction region of esophagus and intestine (D) and intestine (E)) after treatment with 5 g/L *Camellia* seed cake extract for different times. Bars: 10 μm.

**Fig 4:**
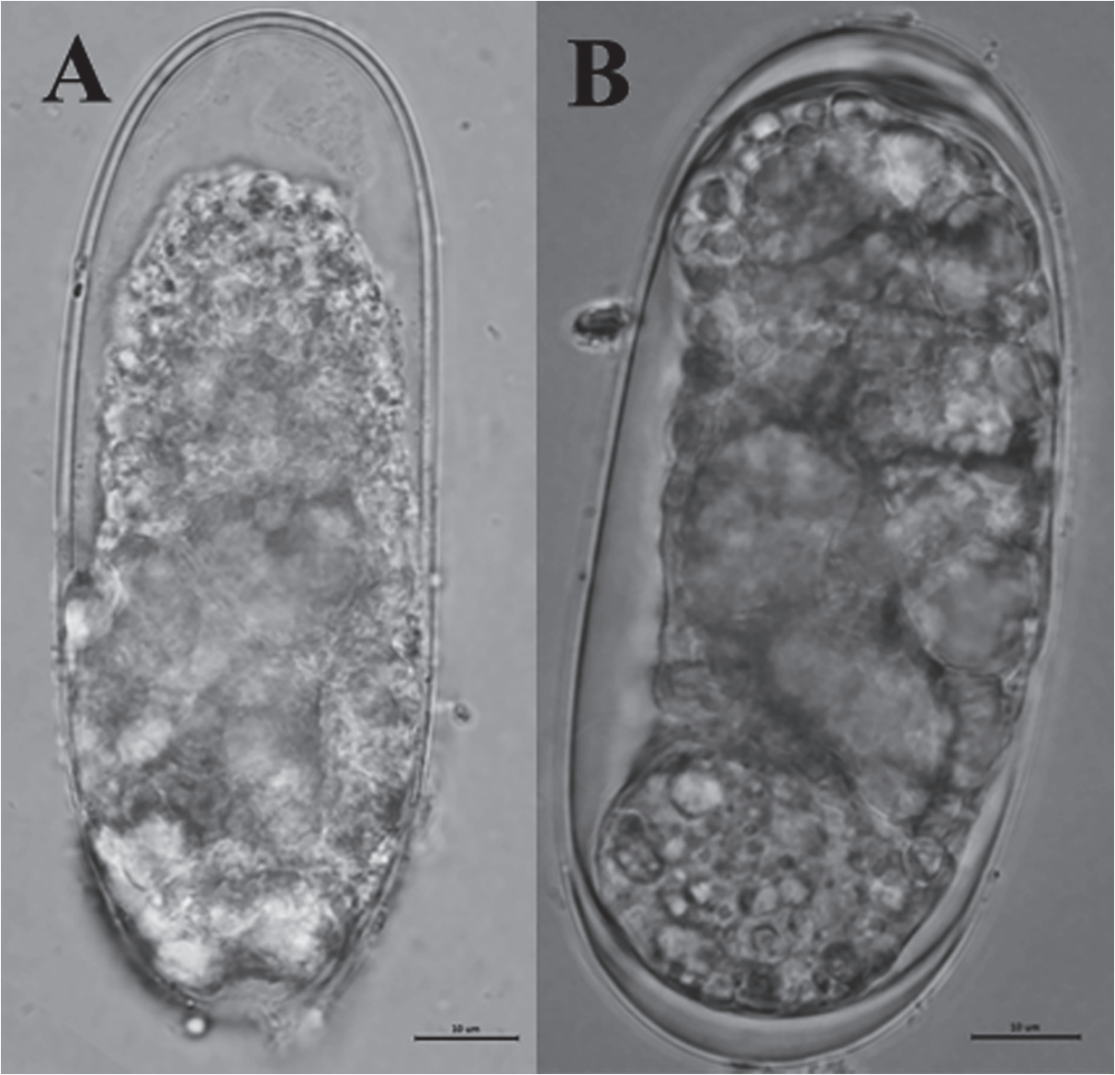
Morphological variations of *M*. *javanica* eggs after treatment with 5 g/L *Camellia* seed cake extract. **The egg treated with 5 g/L extract at 72 h (A) and the egg treated with water control (B).** Bars: 10 μm.

The juvenile had a smooth cuticle and brighter content at 0 h, while the cuticle was crimpled and the intestine was obviously dissolved at 72 h (Fig. 3A). No apparent changes were observed on the anterior part of the body (Fig. 3B) and tail region (Fig. 3C) of J2s. However, the junction region of esophagus (Fig. 3D) and intestine part (Fig. 3E) of J2s was gradually became destroyed as the treatment time increasing. No obvious morphological changes were observed on J2s in water control. The eggs treated with the extracts for 72 h showed the content apparently dissolved and the eggshell layers obviously reduced (Fig. 4A), however, the eggs in water control had a smooth and bright surface and a clear content (Fig. 4B).

### Nematicidal effect of camellia saponin and its molecular weight

**Fig 5:**
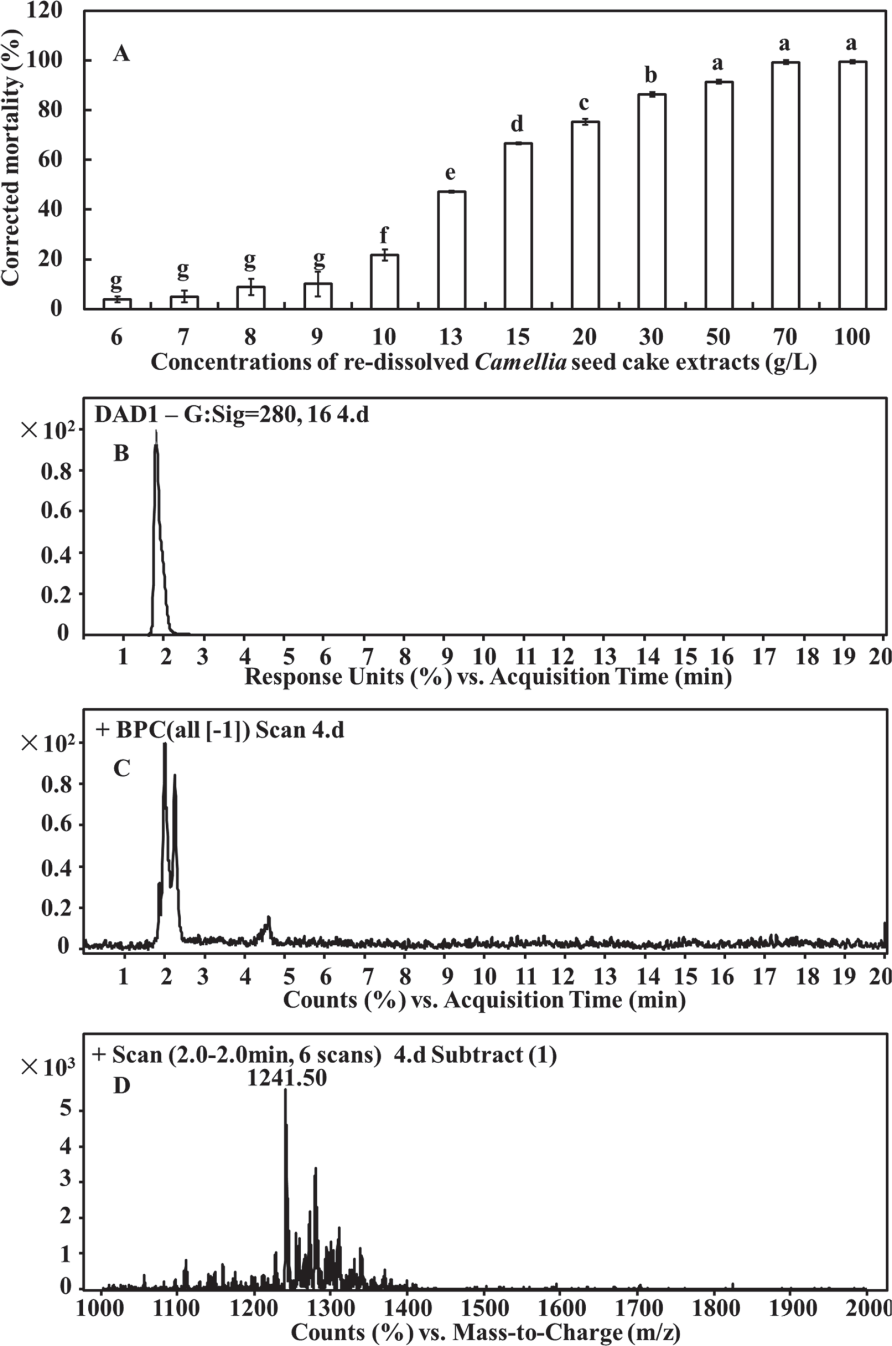
The effects of the re-dissolved extracts on the number of *M*. *javanica* J2s (A) and saponin detected by HPLC-MS (include HPLC profile (B), total ion chromatogram (C) and ESI-MS identification (D)). DAD: Diode-Array Detector; BPC: Base Peak Chromatogram.

In Fig. 5A, nearly 100% nematicidal rates were observed from 100 g/L to 50 g/L. From 50 g/L to 6 g/L, the nematicidal rate showed a continual decline to approximately 3.88%.

One chromatographic peak with retention time of 2.0 min was obtained after HPLC analysis (Fig. 5B). Mass spectrometry analysis detected three major compounds [Rt (min) = 1.85, 2.0, 2.22] in the total ion chromatogram of the mixture ions peak (Fig. 5C). The first and third compound has the same molecular mass as 1001.6 Da [M+H]^+^, however, no evidences were found to support them as saponin homologues (S2 and S3 Figs.). The second compound has a molecular mass of 1241.50 Da [M+H]^+^ (Fig. 5D) in the positive-ion mode, showing that the molecular weight of the compound was 1240.50, which was conjectured as saponin homologues [24–26].

### Nematicidal activity of the volatile compounds in the plate

**Fig 6:**
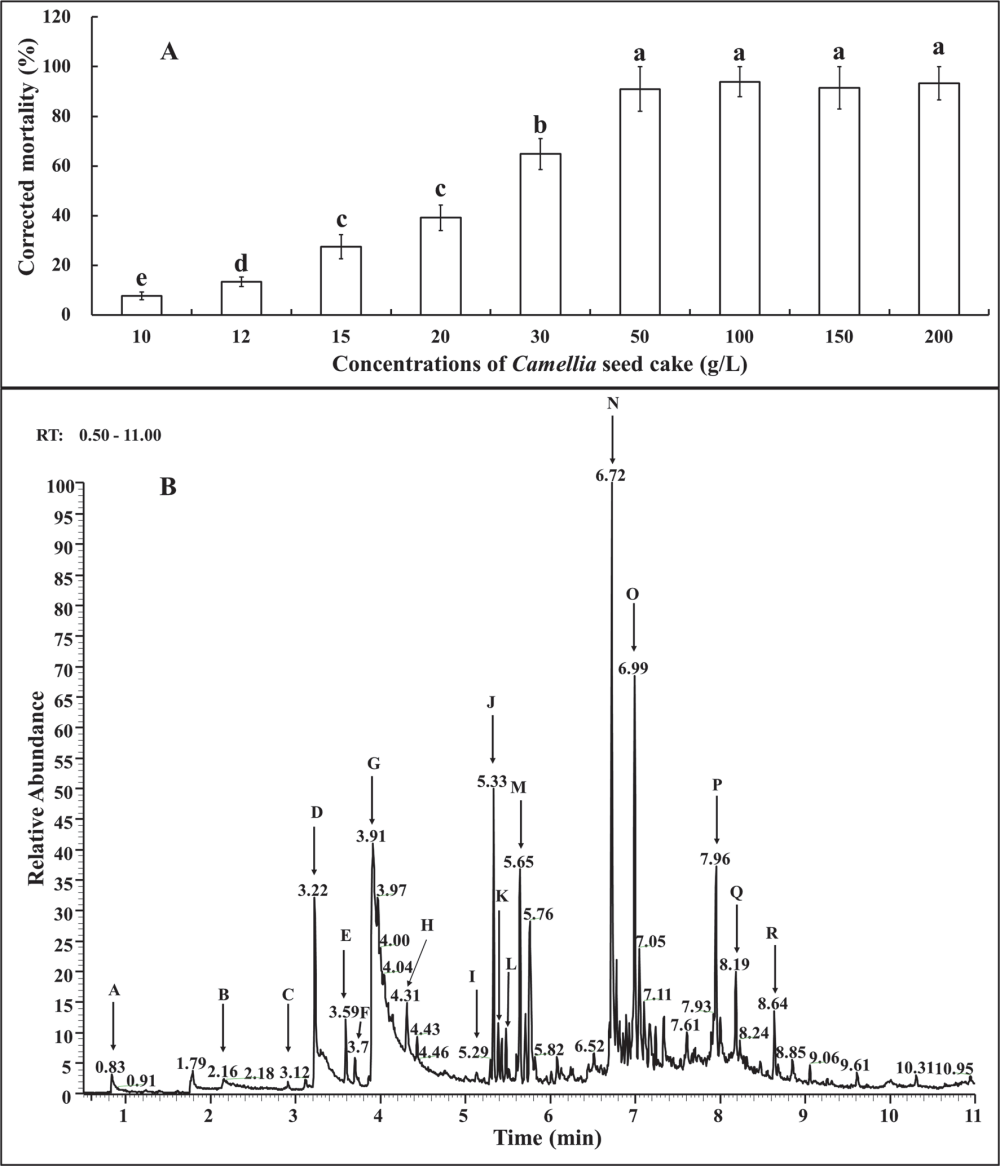
Effects of volatile components (VOCs) from *Camellia* seed cake extract on the *M*. *javanica* J2s (A) and the GC profiles for VOCs produced by the *Camellia* seed cake (B). The compounds corresponding to the retention times 0.83, 2.16, 3.12, 3.22, 3.59, 3.7, 3.91, 4.31, 5.29, 5.33, 5.39, 5.48, 5.65, 6.72, 6.99, 7.96, 8.19, 8.64 were designated as products A, B, C, D, E, F, G, H, I, J, K, L, M, N, O, P, Q and R, respectively.

The effects of VOCs from *Camellia* seed cake extract on the juvenile population of *M*. *javanica* are presented in Fig. 6A. Nearly all the nematodes were killed from 50 g/L to 200 g/L. From 50 g/L to 10 g/L, the nematicidal rate showed a continual decline to approximately 7.56%. In addition, the nematicidal rate was nearly 50% at 20 g/L and 7.56% at 10 g/L (Fig. 6A).

VOCs were further identified by GC-MS. The retention times of the products are summarized in Table 1. According to the NIST library, products A, B, C, D, E, F, G, H, I, J, K, L, M, N, O, P, Q and R shown in Fig. 6B were identified as 1-butanol, 2-methyl-butanoic acid, 1-octen-3-ol, Butylesterbutanoic acid, 2-methylbutanebutyl, 3,3-dimethyloctane, 4-methylphenol, 2-methyl-2-butenoate butyl, 2-bromo dodecane, 2,7,10-trimelthyldodecane, Bcosane, 2,6,10,15-tetramethylheptadecane, 2,6,11-trimethyldodecane, Heptacosane, 2-methyltetradecane, 2,6,10,15-tetramethylheptadecane, 2-methylnanadecane and 3,7,11,15-tetramethyl-2-hexadecen-1-ol, respectively (Table 1). We acquired 8 of the 18 pure compounds; the other 10 were not available for purchase. The effects of the 8 pure compounds on the juveniles of *M*. *javanica* are shown in Table 2. The highest efficiency was observed for 4-methylphenol, which yielded a corrected mortality of 9.50% with an added amount of 3 mg/L.

**Table 1:** Compounds from *Camellia* seed cake identified by GC-MS.

Metabolite	Rt(min)	Chemical name
A	0.83	1-butanol
B	2.16	2-methyl-butanoic acid
C	3.12	1-octen-3-ol
D	3.22	Butylester butanoic acid
E	3.59	2-methylbutanebutyl
F	3.7	3,3-dimethyloctane
G	3.91	4-methylphenol
H	4.31	2-methyl-2-butenoate butyl
I	5.29	2-bromo dodecane
J	5.33	2,7,10-trimelthyldodecane
K	5.39	Bcosane
L	5.48	2,6,10,15-tetramethylheptadecane
M	5.65	2,6,11-trimethyldodecane
N	6.72	Heptacosane
O	6.99	2-methyltetradecane
P	7.96	2,6,10,15-tetramethylheptadecane
Q	8.19	2-methylnanadecane
R	8.64	3,7,11,15-tetramethyl-2-hexadecen-1-ol

**Table 2:** Nematicidal compounds from *Camellia* seed cake identified by GC-MS.

Metabolite	Rt(min)	Chemical name	3000 mg/L	300 mg/L	30 mg/L	3 mg/L
A	0.83	1-butanol	24.26±0.37g	21.50±0.64g	7.18±1.31g	0g
B	2.16	2-methyl-butanoic acid	39.39±0.87e	33.97±0.91d	36.07±5.56c	4.10±0.32b
C	3.12	1-octen-3-ol	100a	45.58±0.18c	40.75±1.07b	4.08±0.11b
D	3.22	Butylester butanoic acid	64.58±2.95b	44.64±0.74c	26.01±1.43d	2.54±0.06de
E	3.59	2-methylbutanebutyl	39.39±0.87e	33.97±1.29d	26.28±0.42d	2.65±0.05d
F	3.7	3,3-dimethyloctane	66.55±0.61b	52.44±1.43b	32.21±0.82c	3.15±0.07c
G	3.91	4-methylphenol	100a	100a	100a	9.50±0.23a
H	5.29	2-bromo dodecane	33.99±2.70f	23.88±1.14f	22.08±1.43de	2.26±0.03e

All values are the mean of five replicates. Numbers following “±” represent the standard errors (SE). Different letters in the same column indicate statistically significant differences at the 0.05 probability level according to the Duncan test.

### Pot experiments

For the first-season, sixty days after transplanting the banana seedlings, the outcomes of each treatment were compared to the control (CK). Treatment A showed the significant differences from CK by increasing the plant height, stem diameter, and the fresh weights of shoots and roots with 51.74%, 31.72%, 36.77% and 18.37%, respectively (Table 3). Treatment A also had the significant differences from CK by decreasing the eggmasses per plant and the eggmasses per gram root with 42.57% and 82.07%, respectively, compared with CK. Treatment B showed the significant differences from CK by increasing the plant height and stem diameter with 27.64% and 24.27%, respectively and decreasing the eggmasses per plant and the eggmasses per gram root with 31.08% and 57.30%, respectively. No significant differences were observed for other growth parameters in Treatment B. Treatment C only increased the stem diameter by 17.19% and decreased the eggmasses per plant by 33.27%. These results show that the application of 5 g/kg of *Camellia* seed cake to the soil effectively promoted banana growth and inhibited *M*. *javanica* infection.

**Table 3:** Effects of application of *Camellia* seed cake on biomass and egg masses densities of banana plants 60 days after transplantation in the pot experiments (first-season).

Treatment	Plant height (cm)	Stem diameter (mm)	Fresh weight of shoots (g)	Fresh weight of roots (g)	Eggmasses per plant	Eggmasses per gram root
CK	18.67±1.53c	25.13±0.26d	158.95±11.95b	113.42±5.50bc	197.33±13.05a	147.61±36.94a
A	28.33±1.53a	33.1±0.2a	217.47±12.90a	134.25±10.46a	113.33±26.31b	26.46±4.34c
B	23.83±3.40ab	31.23±0.15b	161.17±10.92b	122.32±4.75b	136±9.85b	63.03±21.44bc
C	20.4±3.44c	29.45±0.39c	160.82±13.56b	109.24±6.32c	131.67±7.02b	111.29±28.06ab

All values are the mean of five replicates. Numbers following “±” represent the standard errors (SE). Different letters in the same column indicate statistically significant differences at the 0.05 probability level according to the Duncan test.

For the second-season, sixty days after transplanting the banana seedlings, the outcomes of each treatment were compared to the control (CK). Treatment A showed the significant differences from CK by increasing the plant height, stem diameter, the fresh and dry weights of shoots with 26.17%, 20.71%, 52.59% and 66.35%, respectively. Treatment A also had significant differences from CK by increasing the nematode density in soil with 60.15% and decreasing the nematode density in the roots with 28.23%. Treatment B increased the plant height, stem diameter and the dry weight of shoots by 13.73%, 16.79% and 15.48%, respectively. Treatment B also increased the nematode density in soil by 14.18% and decreased the nematode density in roots by 14.29%. Treatment C increased the stem diameter and the dry weight of shoots by 12.49% and 18.93%, respectively, and decreased the nematode density in roots by 9.52% (Table 4). In addition, after identifying the nematodes in the soil using morphology and feeding-habit based classification, the numbers of plant parasites in treatments A and B were significantly lower than in the control, while the numbers of fungivores and bacterivores were greater in treatments A and B than in the control (S1 Table). For the culturable microbes, the numbers of bacteria, fungi and actinomycetes in the three treatments were all significantly higher than in the control (S2 Table).

**Table 4:** Effects of application of *Camellia* seed cake on biomass and nematode densities of banana plants 60 days after transplantation in the pot experiments (second-season).

Treatment	Plant height (cm)	Stem diameter (mm)	Fresh weight of shoots (g)	Dry weight of shoots (g)	Fresh weight of roots (g)	Nematode density in roots (individuals 10 g^−1^ root biomass)	Nematode density in soil (individuals 100 g^−1^ dry soil)
CK	20.9±1.55c	28.83±0.75d	160.71±20.69b	14.74±0.42c	118.91±14.12a	980±65.57a	870±52.92c
A	26.37±1.02a	34.80±0.36a	245.22±4.92a	24.52±0.49a	145.04±29.90a	703.33±65.06c	1393.33±30.55a
B	23.77±1.19b	33.67±0.45b	185.10±12.67b	18.51±1.27b	137.32±19.10a	840±26.46b	993.33±75.72b
C	21.5±0.61c	32.43±0.12c	174.27±5.78b	17.53±0.61b	117.41±18.15a	886.67±20.82b	923.33±41.63bc

All values are the mean of five replicates. Numbers following “±” represent the standard errors (SE). Different letters in the same column indicate statistically significant differences at the 0.05 probability level according to the Duncan test.

For the third-season, similar results for the growth parameters were observed as for the second-season. When compared with the control (CK), treatment A decreased the nematode density in roots by 31.84%. Moreover, treatment A increased the nematode density in soil by 73.30%, and treatment B by 21.87% (Table 5).

**Table 5:** Effects of application of *Camellia* seed cake on biomass and nematode densities of banana plants 60 days after transplantation in the pot experiments (third-season).

Treatment	Plant height (cm)	Stem diameter (mm)	Fresh weight of shoots (g)	Dry weight of shoots (g)	Fresh weight of roots (g)	Nematode density in roots (individuals 10 g^−1^ root biomass)	Nematode density in soil (individuals 100 g^−1^ dry soil)
CK	20.53±0.49c	27.53±0.7d	158.76±21.63b	14.9±0.24c	115.79±13.51a	975.67±48.02a	831.83±46.47c
A	27.77±0.45a	34.6±1.63a	240.18±14.33a	25.01±0.45a	135.67±13.83a	665±36.26c	1441.6±56.26a
B	24.87±1.24b	32.27±0.50b	175.27±6.50b	17.80±0.92b	127.58±13.87a	820±43.44b	1013.8±64.50b
C	20.80±0.72c	29.73±0.55c	163.28±8.31b	17.36±0.57b	118.83±18.14a	871±22.61b	918.62±43.38bc

All values are the mean of five replicates. Numbers following “±” represent the standard errors (SE). Different letters in the same column indicate statistically significant differences at the 0.05 probability level according to the Duncan test.

## Discussion

Root knot nematodes on bananas seriously damage its production and induce the complex diseases caused by the other plant pathogens, therefore exploring a high efficient nematicide with environmental security is urgently needed. In this study, the effects of *Camellia* seed cake on controlling *M*. *javanica* were evaluated and its possible mechanisms for nematicidal activities were elucidated. *M*. *javanica* has already been reported to be the dominant banana pathogen in Pakistan [27]. It has also been reported to be the dominant pathogen for almost all of the world’s major crop plants, including papaya, potato, peanut, tomato, and rootstocks [28–31]. The populations isolated from banana roots in Hainan Province, China were mainly identified as *M*. *javanica* based on morphological and molecular characteristics, which was confirmed to be the dominant species of banana root-knot nematodes in our research.

*Camellia* seed cake acts as a non-conventional fertilizer and has been reported to effectively suppress plant-parasitic nematodes [32]. In this study, more than 50% of *M*. *javanica* J2s were killed at *Camellia* seed cake extract concentrations above 2 g/L, and at these concentrations, half of the *M*. *javanica* eggs could not be hatched. The results proved the potential nematicidal activity of *Camellia* seed cake, which is similar to those of Wang et al. [33], who reported that Camellia plant extracts had strong nematicidal activity on *B*. *xylophilus* and *M*. *incognita*. After treatment with *Camellia* seed cake extracts, the eggs of *M*. *javanica* were gradually dissolved, and the intestine of the juveniles gradually became indistinct in our observation. Similar symptoms have been reported that J2s and eggs of *M*. *incognita* were damaged after treatment with *Bacillus cereus* X5 [34] or 2, 4-diacetylphloroglucinol, the secondary metabolite of *Pseudomonas fluorescens* CHA0 [35]. These *in vitro* tests indicated the eggs and J2s of *M*. *javanica* could be controlled by the *Camellia* seed cake extract.

Several papers have reported that *Camellia* seed cake contained 15% camellia saponin. Therefore, the nematicidal effect and molecular weight of camellia saponin were investigated in this study. Saponins are steroid or triterpenoid glycosides, which are commonly observed in many plants [36]. Saponins from *Pulsatilla koreana* root or from *Medicago sativa* could control plant-parasitic nematodes [37]. Our results also confirmed that camellia saponin possesses a distinct nematicidal effect. After detection by HPLC-ESI-MS, a compound with a molecular weight of 1240.50 Da was observed. In previous reports, the molecular weight of triterpene saponin from *Lysimachia capillipes* is 1240.61 Da [24], from the roots of *Panax notoginseng*, is 1240.65 Da [25–26]. Therefore, we propose that compound detected in this study was confirmed as saponin.

After soaking, volatile gases produced by *Camellia* seed cake were observed to kill most of the root-knot nematodes. Therefore, the volatile gases produced by *Camellia* seed cake were collected using headspace-SPME and identified by GC-MS. The headspace-SPME represents an excellent, solventless analysis technique that has been applied to identify VOCs, e.g., in blood, viscera samples, urine, and food [38–40]. Through GC-MS, 18 compounds were identified, of which 8 standard compounds were reported for the first time to show nematicidal activities. Among them, 4-methylphenol showed the best efficiency. Our results are similar to those of Li et al. [41], who reported that the volatile compound methyl thiobutyrate has inhibiting effects on *Caenorhabditis elegans* and *M*. *incognita*, and can be further applied to prepare a vermifuge from its precursor substance. Most likely, the nematicidal effect of *Camellia* seed cake is due to the production of both volatile and non-volatile inhibitory compounds.

In our three seasons of pot experiments using sterilized or crude soils, the application of *Camellia* seed cake significantly suppressed *M*. *javanica* and promoted plant growth. We speculate that the growth promotion may be attributable to the suppression of the harmful nematode *M*. *javanica* and the nutrient content of the *Camellia* seed cake. Our results agree with previous reports of effective suppression of harmful nematodes by nematicidal agents from plant sources in pot and field experiments. Four medicinal plants, *Azadirachta indica*, *Calotropis procera*, *Datura stramonium* and *Tagetes erecta* had nematicidal effects against *M*. *incognita* [42], while the aqueous and ethanol extracts from leaves, stem, bark and fruit of *Eucalyptus* sp., against *M*. *javanica* [43]. The extracts of *Eucalyptus* sp not only reduced egg hatching and increased J2s mortality as exposure time increased, but also significantly increased the shoot length, shoot weight, root length and root weight [43], which showed the similar nematicidal activities and plant promotion as that of the *Camellia* seed cake.

After morphological observation at the end of the second-season pot experiment, the number of plant parasites in the *Camellia* seed cake treatments was lower than in the control, while the density of total nematodes and other functional groups showed the opposite tendency. This result may be caused by the fact that *Camellia* seed cake application increased the number of bacteria, fungi and actinomycetes, which in turn increased the number of total nematodes, especially bacterivores, fungivores and omnivores. Our results are similar to those of González [44], who showed that situ ingestion rates of fluorescently labeled bacteria could estimate the active bacterivores in natural aquatic systems. Remén [45] showed that ectomycorrhizal fungi constituted an important food source for fungivorous soil fauna and may be a factor regulating these faunal communities. Moreover, promotion of plant growth by bacterial-feeding nematodes has been previously reported [46–47]. The effects of bacterial-feeding nematodes may be another explanation of the growth-promotion effect observed in the pot experiments. Therefore, we proposed that the alteration of nematode populations and different functional groups is a new mechanism for promoting plant growth by the application of *Camellia* seed cake. However, further studies need to be provided to support this hypothesis.

## Conclusions

The harmful root-knot nematode *M*. *javanica* was identified in this study and confirmed to be the dominant species on banana. The *Camellia* seed cake extracts effectively killed J2s of *M*. *javanica* and suppressed the hatching rate of eggs. Nematicidal compounds produced by *Camellia* seed cake, including saponins and VOCs, effectively killed J2s. In pot experiments of banana seedlings, application of *Camellia* seed cake not only suppressed *M*. *javanica* but also promoted plant growth. The direct biocontrol efficiency of *Camellia* seed cake in field experiments requires further study.

## Supporting Information

S1 Fig The split plates (Petri dish with vents, Greiner company, Germany) with one side tiled with 5 mL sterile ultrapure water with different concentrations of *Camellia* seed cake (10, 12, 15, 20, 30, 50, 100, 150, 200 g/L) or pure standard substances, and the other side held 5 mL of ultrapure water with 300 individuals of freshly hatched juveniles of *M*. *javanica*. (TIF)

S2 Fig ESI-MS identification of the compound with Rt (min) of 1.85. (TIF)

S3 Fig ESI-MS identification of the compound with Rt (min) of 2.22. (TIF)

S1 Table Effects of application of camellia cake on nematode fauna for 60 days after transplanting in the pot experiment. (DOC)

S2 Table Effects of application of camellia cake on microbe population for 60 days after transplanting in the pot experiment. (DOC)
