# Supplementary figures and images for: The Nematicidal Effect of Camellia Seed Cake on Root-Knot Nematode Meloidogyne javanica of Banana
Source: PLoS One. 2015 Apr 7;10(4):e0119700. doi: 10.1371/journal.pone.0119700 (PMC4388532; doi:10.1371/journal.pone.0119700)

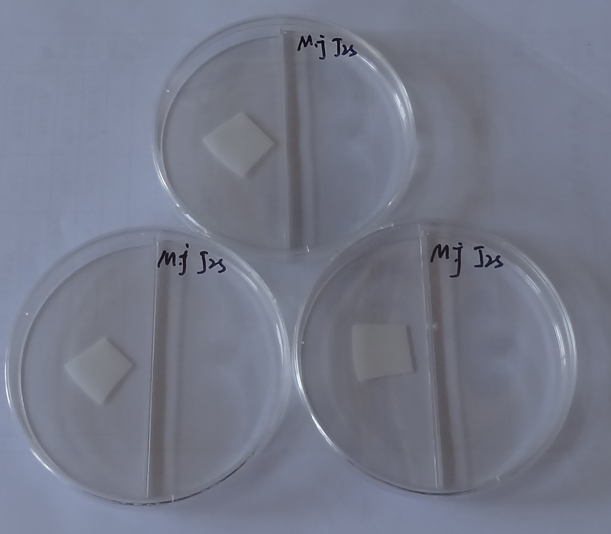

Supplement: S1 Fig — (TIF) [file pone.0119700.s001.tif]

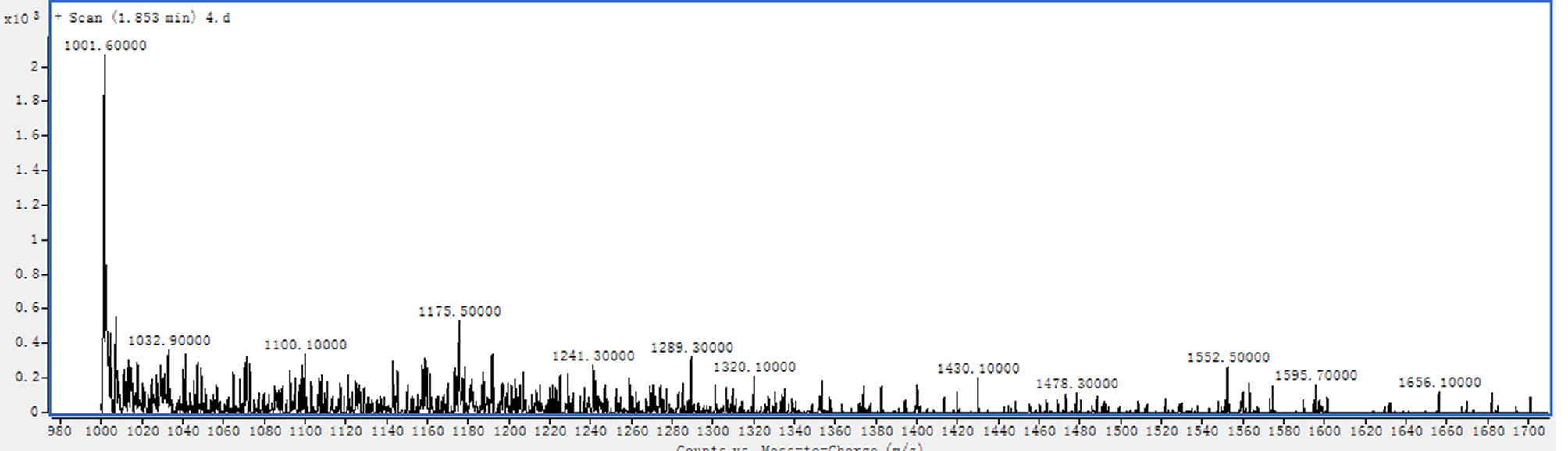

Supplement: S2 Fig — (TIF) [file pone.0119700.s002.tif]

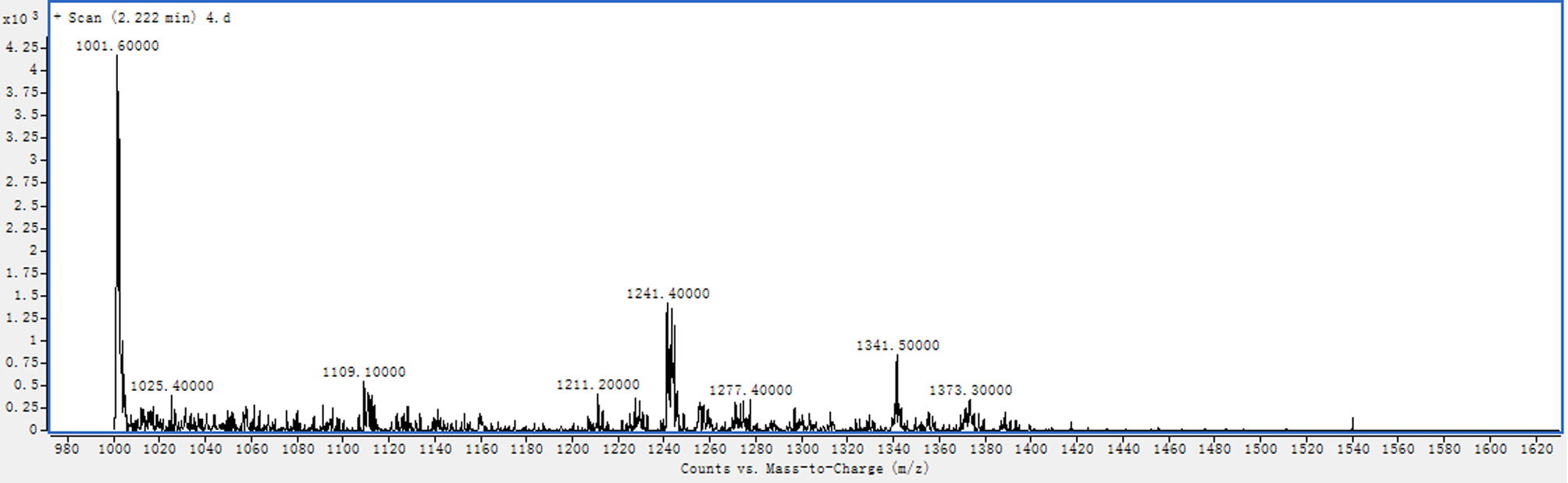

Supplement: S3 Fig — (TIF) [file pone.0119700.s003.tif]
