# Supplementary material for: The Nematicidal Effect of Camellia Seed Cake on Root-Knot Nematode Meloidogyne javanica of Banana
Source: PLoS One. 2015 Apr 7;10(4):e0119700. doi: 10.1371/journal.pone.0119700 (PMC4388532; doi:10.1371/journal.pone.0119700)
Supplement: S1 Table — (DOC) [file pone.0119700.s004.doc]

Table S1 Effects of application of camellia cake on nematode fauna for 60 days after transplanting in the pot experiment

| Nematode fauna | | At the end of the experiment | | | |
| --- | --- | --- | --- | --- | --- |
|  |  | CK | A | B | C |
| Plant–parasites | *Meloidogyne* | 502±30.44a | 348±7.64d | 420±32.11b | 487±21.56a |
|  | *Helicotylenchus* | 67±4.00a | 0±0c | 0±0c | 49±2.08b |
|  | *Ditylenchus* | 100±5.60a | 50±1.22d | 72±5.51c | 83±3.78b |
| Fungivores | *Aphelenchoides* | 33±2.00c | 101±3.53a | 42±3.21b | 33±2.00c |
| Bacterivores | *Mesorhabditis* | 33±2.00d | 348±7.64a | 76±5.75b | 53±2.52c |
|  | *Cephalobus* | 67±3.99d | 403±12.58a | 226±20.09b | 135±6.29c |
|  | *Prismatolaimus* | 33±2.00d | 101±3.53a | 76±5.75b | 47±2.21c |
|  | *Acrobeloides* | 36±2.00c | 50±1.22b | 76±5.75a | 35±1.70c |
| Nematode densities (individuals 100 g^−1^ dry soil) | | 870±52.92c | 1393.33±30.55a | 993.33±75.72b | 923.33±41.63bc |
