# Supplementary material for: The Nematicidal Effect of Camellia Seed Cake on Root-Knot Nematode Meloidogyne javanica of Banana
Source: PLoS One. 2015 Apr 7;10(4):e0119700. doi: 10.1371/journal.pone.0119700 (PMC4388532; doi:10.1371/journal.pone.0119700)
Supplement: S2 Table — (DOC) [file pone.0119700.s005.doc]

Table S2 Effects of application of camellia cake on microbe population for 60 days after transplanting in the pot experiment

| Treatment | Bacteria (×10^8^ CFU·g^-1^ dry soil) | Fungi (×10^4^ CFU·g^-1^ dry soil) | Actinomycetes (×10^5^ CFU·g^-1^ dry soil) |
| --- | --- | --- | --- |
| CK | 0.35±0.03d | 0.95±0.17d | 1.51±0.16d |
| A | 11.67±1.1a | 9.13±1.01a | 9.6±0.4a |
| B | 6.47±0.83b | 4±0.53b | 6.87±0.31b |
| C | 4.47±0.51c | 2.4±0.35c | 2.07±0.42c |
